# Multi‐modal MRI reveals changes in placental function following preterm premature rupture of membranes

**DOI:** 10.1002/mrm.29483

**Published:** 2022-10-18

**Authors:** Jana Hutter, Paddy J. Slator, Carla Avena Zampieri, Megan Hall, Mary Rutherford, Lisa Story

**Affiliations:** ^1^ Centre for the Developing Brain King's College London London United Kingdom; ^2^ Centre for Medical Engineering King's College London London United Kingdom; ^3^ CMIC, University College London London United Kingdom; ^4^ Institute for Women's and Children's Health, King's College London London United Kingdom; ^5^ Fetal Medicine Unit, St Thomas' Hospital London United Kingdom

**Keywords:** Diffusion MRI, pregnancy, preterm birth, Relaxometry

## Abstract

**Purpose:**

Preterm premature rupture of membranes complicates up to 40% of premature deliveries. Fetal infection may occur in the absence of maternal symptoms, delaying diagnosis and increasing morbidity and mortality. A noninvasive antenatal assessment of early signs of placental inflammation is therefore urgently required.

**Methods:**

Sixteen women with preterm premature rupture of membranes < 34 weeks gestation and 60 women with uncomplicated pregnancies were prospectively recruited. A modified diffusion‐weighted spin‐echo single shot EPI sequence with a diffusion preparation acquiring 264 unique parameter combinations in < 9 min was obtained on a clinical 3 Tesla MRI scanner. The data was fitted to a 2‐compartment $T_{2}^{*}$‐intravoxel incoherent motion model comprising fast and slowly circulating fluid pools to obtain quantitative information on perfusion, density, and tissue composition. *Z* values were calculated, and correlation with time from between the rupture of membranes and the scan, gestational age at delivery, and time between scan and delivery assessed.

**Results:**

Placental $T_{2}^{*}$ was significantly reduced in preterm premature rupture of membranes, and the 2‐compartmental model demonstrated that this decline is mainly linked to the perfusion component observed in the placental parenchyma. Multi‐modal MRI measurement of placental function is linked to gestational age at delivery and time from membrane rupture.

**Conclusion:**

More complex models and data acquisition can potentially improve fitting of the underlying etiology of preterm birth compared with individual single‐contrast models and contribute to additional insights in the future. This will need validation in larger cohorts. A multi‐modal MRI acquisition between rupture of the membranes and delivery can be used to measure placental function and is linked to gestational age at delivery.

## 1 INTRODUCTION

Preterm premature rupture of membranes (PPROM) complicates up to 40% of deliveries less than 37 weeks gestation.^1^ Neonatal morbidity, including sepsis, cystic periventricular leukomalacia, intraventricular hemorrhage, and later development of cerebral palsy, are significantly higher among pregnancies with PPROM complicated by infection, confirmed by histopathological evidence of chorioamnionitis postdelivery.^2^ Fetal infection can occur in the absence of overt clinical signs in the mother^3^ and is therefore often a retrospective diagnosis postdelivery with acute inflammatory and vascular lesions found in over 20% to 40% of assessed placentas.^4^, ^5^

The current retrospective diagnosis limits optimal timing of antenatal interventions such as corticosteroids or delivery. Current clinical practice is to delay delivery until 37 weeks in the absence of overt signs of infection.^6^ There is a pressing need for noninvasive antenatal assessment of early signs of placental inflammation that may be indicative of fetal infection during this prolonged time window. Current biomarker techniques proposed include the maternal neutrophil–lymphocyte ratio,^7^ but this is not routinely used in clinical practice and evidence for benefit is still outstanding.

Blood reaches the spiral arteries from the uterine arteries, allowing a continuous and slow supply of highly oxygenated maternal blood to the placenta. Blood enters the intervillous spaces, bathing the fetal vasculature contained in villous trees. Fetal blood resides in hairpin‐like villous capillaries without mixing with the maternal blood. Inflammatory responses to maternal infection are associated with an increased concentration of neutrophils and cytokines in the placenta and increased deposition of placental fibrin in later stages.^3^ Changes in both the molecular properties of placental tissue as well as in the microstructure are thus expected. Multi‐modal MRI techniques are ideally suited to assess the structural and functional properties of tissues and can therefore play a crucial role in helping to discriminate between acute and chronic phases of inflammation in vivo. MRI‐based techniques have been successfully used in inflammatory processes in the liver^8^ and heart.^9^

Among the most promising placental MRI techniques are $T_{2}^{*}$ relaxometry and diffusion MRI. $T_{2}^{*}$ focuses on the molecular properties of tissue, especially exploiting the blood–oxygen level dependency (BOLD), which correlates the concentration of deoxygenated hemoglobin with the $T_{2}^{*}$ values. Typically used multi‐echo gradient‐echo sequences sample data at multiple TEs to allow mono‐exponential fitting to obtain $T_{2}^{*}$ maps.

Diffusion MRI can reveal microstructural characteristics by fitting models to data acquired using gradients of different strength and direction (*b* value/*b* vector).

One commonly used model is the intravoxel incoherent motion (IVIM) model, exploiting the bi‐phase behavior of the MRI signal decay with growing *b* value to obtain a pseudo‐diffusivity—associated with water in perfusing blood—and a diffusivity value—associated with water diffusing in tissue. The placenta's unique vascular structure—in particular, the fact that maternal blood slowly perfuses through the intra‐villous space—complicates interpretation of IVIM model parameters. It is possible that a proportion of perfusing maternal blood will psuedo‐diffuse slowly enough to show up in the diffusion compartment.

Both $T_{2}^{*}$ and diffusion MRI have been successfully used for placental assessment in vivo to visualize and quantify microstructural differences in conditions such as chronic hypertension, preeclampsia,^10^, ^11^, ^12^ and fetal growth restriction.^13^, ^14^ There has been recent focus on the IVIM model, showing increased perfusion fraction inside the placental parenchyma compared to control cases,^15^ reduced perfusion fraction in placentae with evidence of fetal vascular malperfusion,^16^ and reduced birth weight^17^, ^18^ as well good robustness of the placental perfusion fraction measurements.^19^

In addition to studies focusing on $T_{2}^{*}$ or diffusion individually, joint modeling and acquisition approaches for diffusion‐relaxometry experiments have been performed. Analytic techniques include a 2‐compartment model, which has been successfully used in fetal growth restriction,^20^ as well as 2‐compartment models with Laplacian approaches employed to assess preeclampsia.^21^ These more complex models reveal distinct structural and functional phenotypes. Finally, new acquisition techniques have been proposed, allowing the acquisition of data necessary for such combined experiments in 1 scan. This provides a more efficient and approach to motion.^21^, ^22^, ^23^

Such combined models potentially fit the complex physiological processes expected in in‐utero infection and inflammatory responses well but have not been deployed to date. In the context of PPROM, fetal MRI poses additional challenges. The reduced amount of amniotic fluid changes the properties of the magnetic field and thus incurs additional artifacts and changes the contrast surrounding the placenta, leading to more difficult segmentation. So far, MRI in PPROM was limited to volumetric assessment of the brain,^24^ lung,^25^ and thymus,^26^ as well as to the described $T_{2}^{*}$ and diffusion techniques in isolation, showing decreased mean $T_{2}^{*}$ and ADC.^27^

This study presents an efficient multimodal $T_{2}^{*}$‐diffusion acquisition with dedicated multi‐compartmental combined diffusion‐relaxometry analysis to study differences in placentas affected by PPROM compared to those from control pregnancies.

## 2 METHODS

Women at high risk of preterm delivery were prospectively recruited from St Thomas' Hospital (London, UK) between April 2019 and March 2022 as part of 3 ethically approved studies. (Not all eligible women were approached during this time period, only during periods when the study team was recruiting.) Inclusion criteria were gestational age between 20 and 34 weeks and PPROM, confirmed on clinical assessment at vaginal speculum examination recruited from the antenatal ward or maternity assessment unit, or pregnancies considered low risk at the time of study entry. Exclusion criteria were known structural or chromosomal abnormalities, multiple pregnancies (twins, triplets), active labor, maternal inability to give informed consent, pregnancy complications including preeclampsia and fetal growth restriction, and contraindications to MRI such as claustrophobia or a recently sited metallic implant.

Key clinical parameters included maternal age and body mass index at booking, as well as previous medical and obstetric history such as previous term or preterm deliveries, current medication history, date of rupture of membranes, and any treatment received. Furthermore, extensive pregnancy outcome data were obtained, including birth weight, gestational age at delivery, Apgar score, and details regarding any neonatal admissions. The time between membrane rupture and scan and the time between scan and delivery were calculated as displayed in Figure A. Maternal neutrophil–lymphocyte ratio values were obtained from the majority of the PPROM participants within days of the MRI scan but not from the low‐risk participants.

Participants were scanned in supine position on a 3 Tesla Philips Achieva scanner (Philips, Best, Netherlands) using a cardiac 32‐channel coil after informed consent was obtained. Constant life monitoring using an in vivo device assessing heart rate, oxygen saturation, and blood pressure was performed. Frequent verbal interaction was maintained throughout the scan. The scan lasted 60 min, with a break after approximately 30 min for maternal comfort. The acquisition is illustrated schematically in Figure B.

### 2.1 Acquisition protocol

After pilot and calibration scans, T_2_‐weighted turbo spin echo sequences were performed covering the entire uterus in sagittal and coronal plane to maternal habitus as well as at 45‐degree angles sagittal focusing on the fetal brain. The following scanning parameters were used: TR = 25 s, TE = 80 ms, slice thickness of 2.5 mm, slice overlap of 1.25 mm, with a flip angle = 90°.

A B_0_ map covering the entire uterus was acquired, and image‐based shimming focusing on the placenta was performed,^28^ restricted to the placental parenchyma and carefully avoiding maternal bowel and fetal structures. The latter are of particular importance in the absence of amniotic fluid. Data was then acquired with a multi‐slice 2D multi‐echo gradient echo sequence (MEGE) covering the entire uterus (resolution 3 mm isotropic, 5 TE in[18.159] ms, TR = 3 s) in under 1 min, and a modified spin‐echo diffusion‐weighted single‐shot EPI sequence using the thus obtained shim values.^22^ The parameters were chosen such that 3 mm isotropic resolution could be achieved while limiting the acoustic output of the sequence, resulting in FOV = 340 mm × 400 mm × 28 mm, TR = 3.4 sec, SENSE = 2, minimal TE = 78 ms. This approach was taken based on previous work,^29^ with measurements at iso‐center performed for different echo‐spacing values. Three subsequent TEs were chosen by minimizing delay time between subsequent readout trains. This resulted in 4 TEs being chosen as [78, 114, 150, and 186] ms, with the readout length of 36 ms setting the deltas. The diffusion preparation parameters were selected as previously optimized.^30^ They include 3 diffusion gradient directions at *b* = [5, 10, 25, 50, 100, 200, 400, 600, 1200, 1600] s mm; 8 directions at *b* = 18 s mm; 7 at *b* = 36 s mm, and 15 at *b* = 800 s mm. In total, 256 (66*4) unique preparations were acquired. Both MEGE and $T_{2}^{*}$‐diffusion data was acquired in the coronal plane to maternal habitus, resulting in a total acquisition time for both of under 10 min.

### 2.2 Reconstruction, motion correction, and selection of the region of interest

The data was reconstructed using in‐house tools^31^ and motion corrected using Ants.^32^ For the motion correction, a template was constructed from the average of volumes acquired with the lowest TE. Then, registration was performed, resulting in a set of transformations correcting the data from the first echo. These were then applied to the other echoes, exploiting the fact that the data for all echoes for each slice is acquired in < 200 ms, hence not presenting significant motion artifacts. The placental parenchyma was manually segmented on all slices of the second TE of the MEGE and on the motion‐corrected $T_{2}^{*}$‐diffusion dataset, avoiding amniotic fluid on the chorionic plate and maternal vasculature on the basal plate.

Mono‐exponential fitting was performed on the MEGE dataset, resulting in $T_{2}^{*}$ maps (MEGE‐$T_{2}^{*}$) and proton density (MEGE‐S_0_) maps. Analysis of the $T_{2}^{*}$‐diffusion datasets was performed using in‐house python scripts (version 1.0) and our extensions to the dmipy python library for diffusion models.^33^, ^34^ These included both a simpler biexponential model of $T_{2}^{*}$ and ADC decay ($T_{2}^{*}$ ADC model; see Equation 1, where $T_{2}^{*}$ and ADC are the unknowns), and a $T_{2}^{*}$‐IVIM model including slow and fast diffusion in 2 compartments and representing diffusing and perfusing blood within the placenta (IVIM model; see Equation 2).

(1) $$T_{2}^{*}\;\mathrm{ADC}\;\text{model}:\;S\left(T_{E},\;b\right)=S_{0}e^{-\left(T_{E}-T_{\text{Emin}}\right)/T_{2}^{*}}e^{-\text{bADC}}$$

(2) $$\begin{array}{ll} & T_{2}^{*}\text{IVIM}\;\text{Model}:\;S\left(T_{E},\;b\right)\\ & =S_{0}\left[{\mathrm{fe}}^{-\left(T_{E}-T_{E\min}\right)/T_{2\text{fast}}^{*}}e^{-{\mathrm{bD}}^{*}}+\left(1-f\right)e^{-\left(T_{E}-T_{E\min}\right)/T_{2\text{slow}}^{*}}e^{-\text{bADC}}\right], \end{array}$$

whereby T_E_ is the TE; T_Emin_ is the lowest TE acquired; *b* is the *b* value; ADC is the apparent diffusion coefficient; $T_{2}^{*}$ is the effective transverse relaxation time; and S_0_ is the signal at the lowest TE with zero diffusion weighting. The resulting parameters are the $T_{2}^{*}$ of the perfusion compartment $T_{2\;\text{fast}}^{*}$ and of the diffusion compartment $T_{2\;\text{slow}}^{*}$, the pseudo‐diffusivity D* (or ADC1) describing the perfusion, and the ADC in the diffusion compartment ADC (ADC2), as well as the fraction f expressing the percentage of each voxel belonging to the perfusion compartment.

### 2.3 Statistical analysis

Linear regression was performed on all the control cases in python using scipy, resulting in the slope, intercept, *P* value, and R2 score. A *P* value < 0.005 was considered significant. Next, z‐scores were calculated based on the control cohort using the method by DeVore et al. for cross‐sectional cohorts.^35^ The z‐scores for the PPROM cohort were obtained, and the correlation between these and 3 key measures regarding PPROM (gestation at delivery, time between scan and delivery, and time between ROM and scan) were calculated using linear regression.

**Table:** 

**Slope controls**	**R** ^**2**^ **/*P* value**	***P* value from the**
**over gestational age**	**value controls**	***z*‐scores cohort**
	**over gestational age**	**difference**

## 3 RESULTS

Sixteen PPROM participants and 60 healthy control participants were included in this study, and clinical characteristics are given in Supporting Information Figure 1 and Supporting Information Table 4, highlighting the lower gestational age at delivery (29 vs. 40 weeks gestational age) as expected, the maintained birth weight centile (59 vs. 60), similar age, and slightly increased body mass index (24 vs. 21) in the PPROM group, as well as decreased time between scan and delivery in the PPROM group.

**FIGURE 1:**
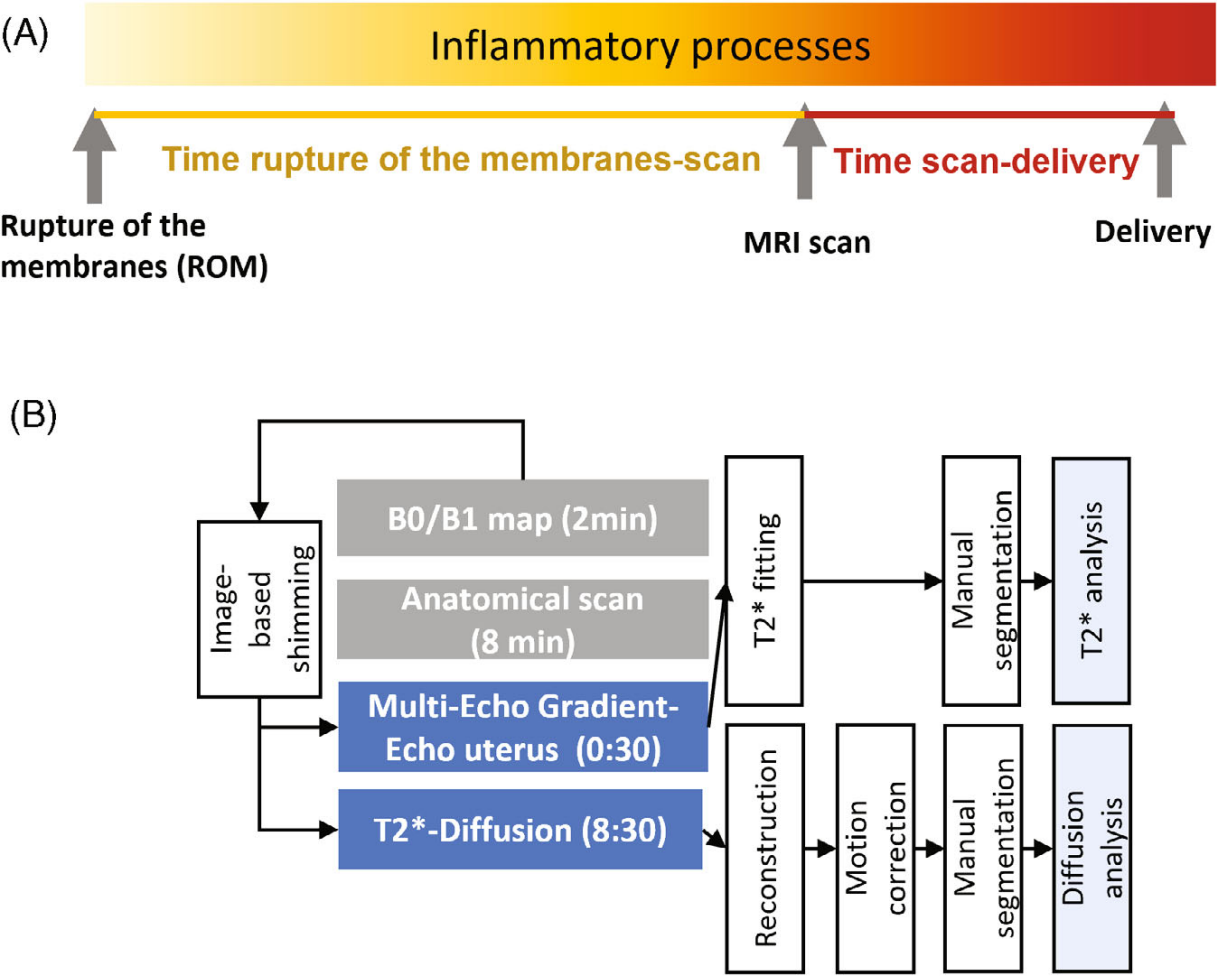
Overview of study showing (A) the time course of investigations, and (B) the MRI protocol and processing schemata describing the data flow

**FIGURE 2:**
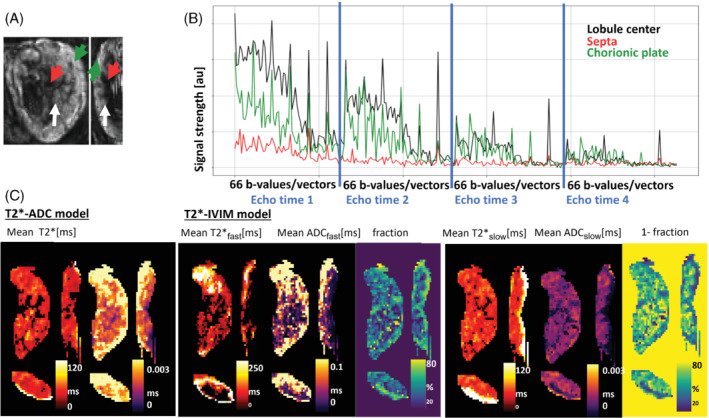
Boxplots for all considered quantities. Top row: $T_{2}^{*}$, ADC from $T_{2}^{*}$‐ADC fit, $T_{2}^{*}$ from $T_{2}^{*}$‐ADC fit, ADC1 from IVIM fit. Bottom row: ADC2 from IVIM fit, $T_{2}^{*}$ 1 from IVIM fit, $T_{2}^{*}$ 2 from IVIM fit, fraction from IVIM

Figure A–2B display the signal evolution curves for 1 exemplary case over all dynamics, varying TE while also varying the diffusion preparation for each echo as outlined above for 3 depicted regions: lobule center, septa, and chorionic plate.

**FIGURE 3:**
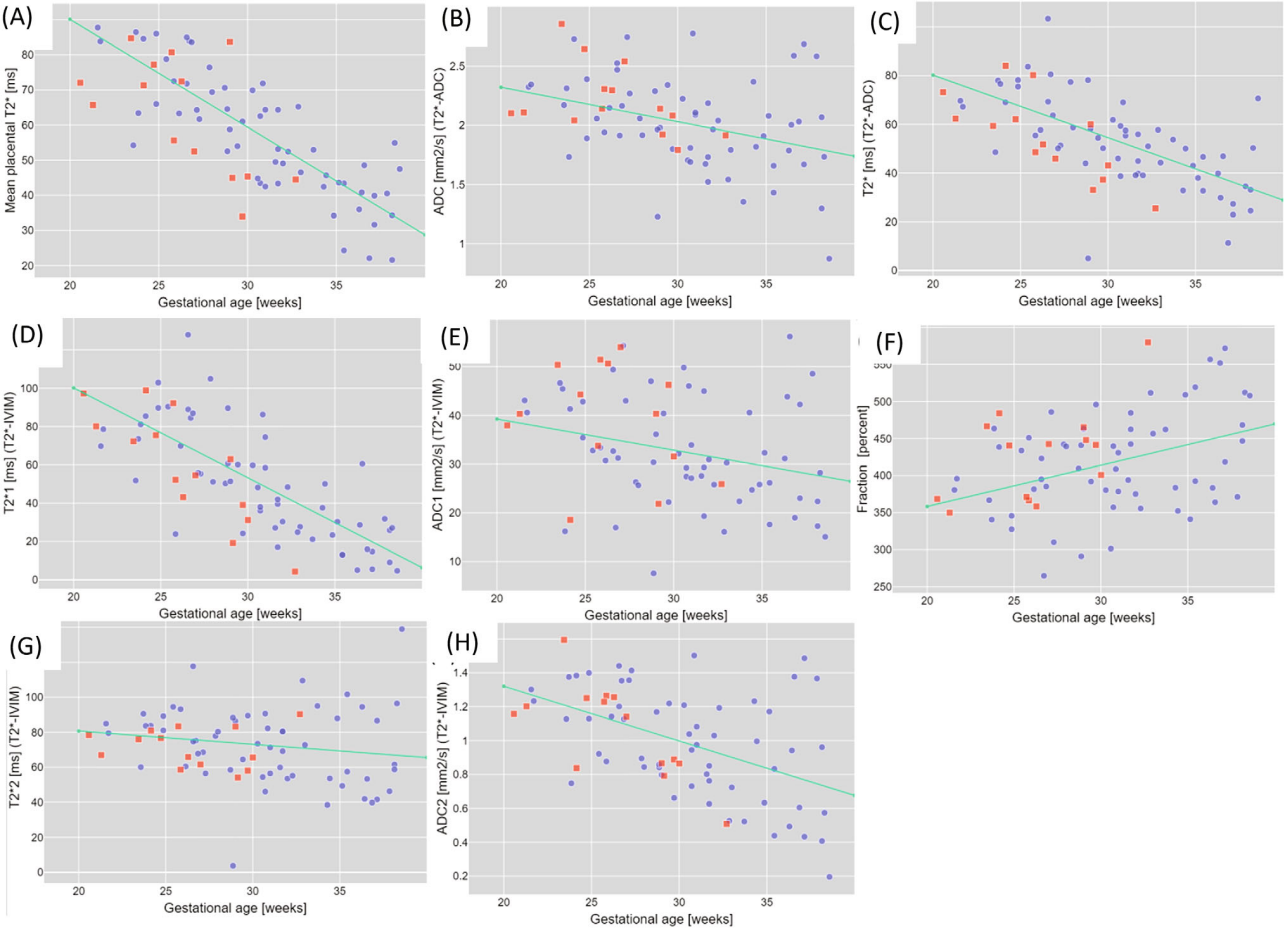
(A) Selected regions of interest in the central lobule areas (indicated by white arrow), the septa (indicated by red arrow), and the basal plate (indicated by green arrow) together with (B) signal evolution over all acquired data points. (C) Quantitative maps obtained using $T_{2}^{*}$‐ADC and the $T_{2}^{*}$‐IVIM model, displaying $T_{2}^{*}$ and ADC map; the $T_{2}^{*}$, ADC map and tissue fraction map of the perfusion compartment; and the $T_{2}^{*}$, ADC, and fraction map of the diffusion compartment from the IVIM (intravoxel incoherent motion) model

Parametric maps for both considered models for the $T_{2}^{*}$‐ADC and the $T_{2}^{*}$‐IVIM models for 1 case are shown in Figure C, displaying reformatted views in all 3 orientations for each measure. The perfusion compartment thus dominates in the centers of the lobules where the spiral arteries enter the placental parenchyma as well as closer to the chorionic plate. The slow diffusion compartment dominates in the intervillous spaces but distant from the spiral arteries inflow.

The quantitative results for all 3 models in Figure 3 display the changes over gestation and between the cohorts; these formed the basis for the calculated *z*‐scores. Thereby, significant correlation for the control cohort over gestational age could be found for $T_{2}^{*}$, $T_{2}^{*}$ from $T_{2}^{*}$‐ADC, $T_{2\;\text{fast}}^{*}$ from $T_{2}^{*}$‐IVIM, and ADC_slow_ from $T_{2}^{*}$‐IVIM (all < 0.005), as well as for the perfusion fraction. No correlation with gestational age was found for the PPROM cohort (see Table 1).

**FIGURE 4:**
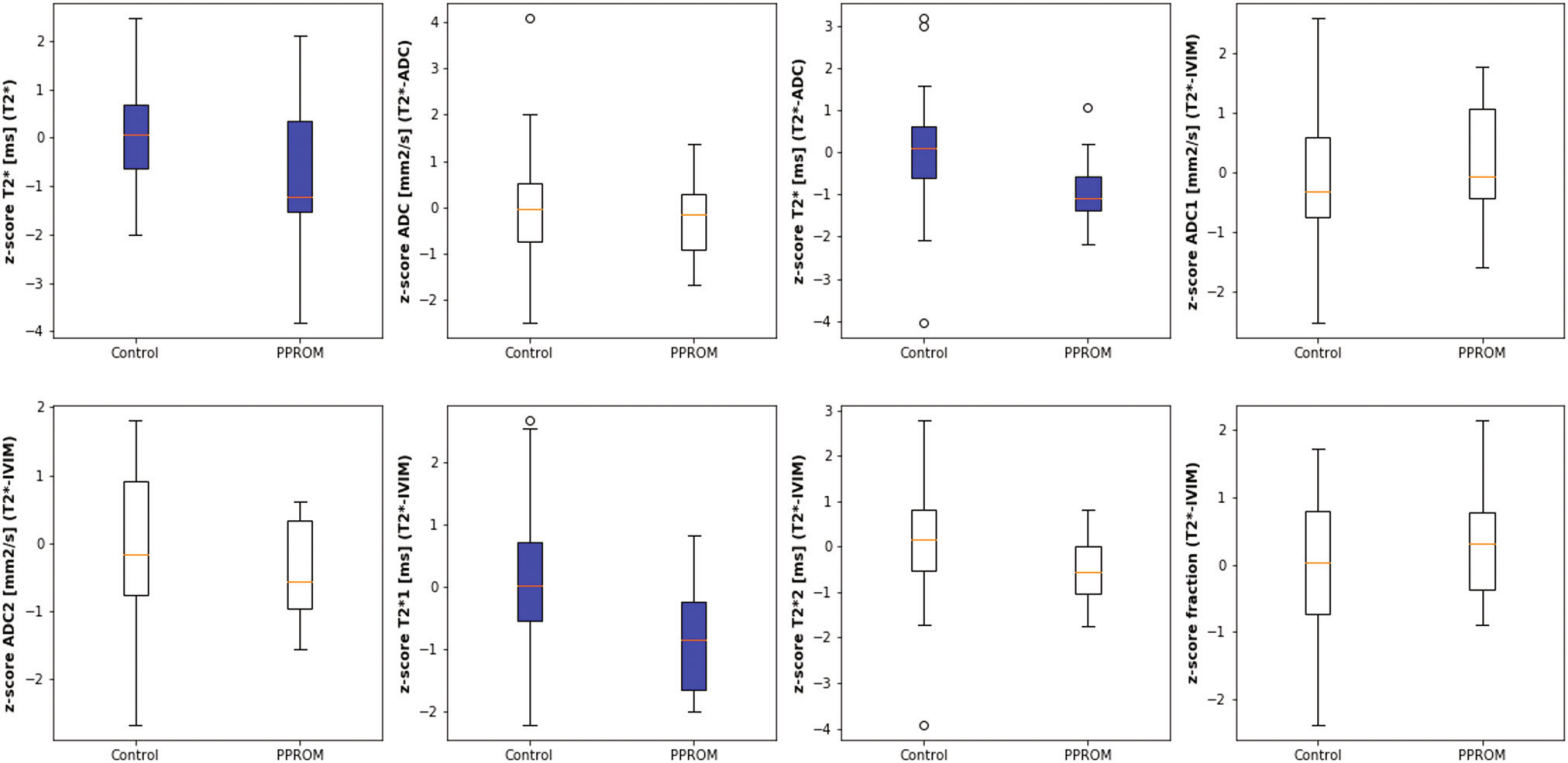
Quantitative results for the $T_{2}^{*}$ data (A) for the $T_{2}^{*}$ (B), and ADC (C) for the $T_{2}^{*}$‐ADC fit and for the $T_{2}^{*}$‐IVIM fit (D, E, F, G, H, I) The control subjects are marked with blue squares and the PPROM cases with red dots; the green line represents the regression line obtained for the controls PPROM, preterm premature rupture of membranes.

**TABLE 1:** Quantitative results for the control values over gestation and the *P* values for the *z*‐score cohort difference

*N* = 16 PPROM *N* = 60 controls	Slope controls over Gestational Age	R^2^/*P* value controls over Gestational Age	*p*‐value from the *z*‐scores cohort difference
$T_{2}^{*}$	−3.004 ms/week	0.81/**<0.005**	0.014
**ADC from** $T_{2}^{*}$ **‐ADC**	−0.035	0.37/**<0.005**	0.429
$T_{2}^{*}$ **from** $T_{2}^{*}$ **‐ADC**	−2.615 ms/week	0.65/**<0.005**	**<0.005**
**ADC1 from** $T_{2}^{*}$ **‐IVIM**	−0.764	0.31/0.016	0.589
**ADC2 from** $T_{2}^{*}$ **‐IVIM**	−0.033	0.49/**<0.005**	0.147
$T_{2}^{*}$ **1 from** $T_{2}^{*}$ **‐IVIM**	−4.791 ms/week	0.57/**<0.005**	**<0.005**
$T_{2}^{*}$ **2 from** $T_{2}^{*}$ **‐IVIM**	−0.671 ms/week	0.16/0.214	0.069
**Fraction perfusion**	5.726%/week	0.39/**<0.005**	0.394

*Note*: The bold indicates cut‐off for significance at p < 0.005. Abbreviations: IVIM, intravoxel incoherent motion; PPROM, preterm premature rupture of membranes.

The *z*‐scores for the PPROM cases were thereby significantly reduced for $T_{2}^{*}$ (*P* < 0.05) (Figure A), $T_{2}^{*}$ from $T_{2}^{*}$‐ADC (*P* < 0.005) (Figure C), and for the $T_{2\;\text{fast}}^{*}$ (perfusion compartment) (*P* < 0.005) (Figure E). The $T_{2\;\text{slow}}^{*}$ (diffusion compartment) showed a trend toward significant reduction (*P* = 0.069). The *z*‐scores for all ADC values did not show a significant difference (*P* = 0.42 for ADC from $T_{2}^{*}$‐ADC, *P* = 0.59 and 0.15 for ADC for perfusing and diffusing compartment, respectively), neither did the perfusion fraction (*P* = 0.39).

Next, the correlation between the *z*‐scores for all the quantitative measures and 3 key measures regarding PPROM, gestation at delivery, time between scan and delivery, and time between ROM and scan were investigated. The results show significant correlations for the ADC in the compartment mainly dominated by the perfusion compartment, that is, with the time of delivery, the time since ROM and between the $T_{2\;\text{slow}}^{*}$ (diffusing compartment), and time between scan and delivery (*P* < 0.05).

## 4 DISCUSSION

This study demonstrated the first results of a simultaneous diffusion‐relaxometry MRI acquisition and multi‐compartmental analysis in pregnant women with PPROM. Reduced mean placental $T_{2}^{*}$ was significantly correlated with PPROM both for the values obtained with a $T_{2}^{*}$ model alone (*P* < 0.05), a $T_{2}^{*}$‐Diffusion model (*P* < 0.005), and the more complex IVIM model for the perfusion compartment (*P* < 0.005). The latter bicompartmental model result and stronger level of significance demonstrated in this study give evidence that the decay is driven by the fast‐flowing perfusion compartment, mostly attributed to maternal inflowing blood. Interestingly, the perfusion fraction, describing how many voxels are predominantly perfusing fast‐flowing blood, trends toward higher values for PPROM. This could potentially correspond to an increase in neutrophils and cytokines in the maternal arteries toward the intervillous spaces as described in histopathological studies of PPROM placentas.^3^ However, the perfusion compartment dominated also on the fetal chorionic plate. Potentially this could hint toward an increase in fetal blood supply, in itself a sign of fetal inflammatory response. Further investigations would however be required to assert this. A larger cohort would be required to investigate these spatial differences in more detail. The use of such more complex models might thus allow us in the future to work toward a better understanding of the pathophysiology underlying PPROM and open new focused avenues to understand the observed differences.

We observed in our data also an increase in perfusion fraction for healthy controls over gestation, in accordance with some previous studies.^15^, ^36^ An increase in perfusion in the intervillous space would be in line with studies using ultrasound bubbles showing an increase over gestation,^37^ as well as an increase in blood flow to the feto‐placental compartment as the pregnancy progresses.

This study, however, has a number of important limitations. These include the number of subjects; recruiting pregnant women with PPROM is a significant challenge both due to the high level of clinical surveillance required and the unpredictable interval between rupture of membranes and delivery. This complicates the scheduling of an MRI scans. This limitation furthermore makes longitudinal within‐subject follow‐up very complicated and thus hampers our ability to study the progression of inflammatory processes. However, as outlined in the introduction, the clinical utility of MRI may be in cases with diagnostic uncertainty where no maternal signs of infection are apparent, and deferral of delivery until 37 weeks is often advocated.

Next, the choice of the region of interest for the analysis is critical. This study has focused on the placental parenchyma, carefully avoiding maternal tissues, for example, in the basal plate. This was chosen with the aim to understand both the placental involvement and to avoid inaccuracy in the segmentation of the thin maternal tissues. Future studies could focus on regions of interest in the basal plate and inclusion of perfusion techniques, measuring noninvasively the inflow of blood into the tissue directly.^38^, ^39^, ^40^

## 5 CONCLUSION

Data was successfully acquired in a challenging group of patients and analysis revealed the first correlations between quantitative modal results from the placenta and the time between rupture of the membranes and delivery. This combination of efficient data acquisition and multi‐compartmental modeling allows a comprehensive study of microstructure and function and can thus potentially play a role in assessing the impact of inflammation and infection on placental function. Future work will link to other longitudinal ultrasound and serum markers to further enhance information about these pregnancies and thus further optimize delivery and intervention.

## FUNDING INFORMATION

This work was supported by the Welcome Trust, the National Institutes of Health (NIH) Human Placenta Project, grant 1U01HD087202‐01 (Placenta Imaging Project [PIP]); Welcome Trust Collaboration in Science, grant [WT201526/Z/16/Z]; a Wellcome Trust Sir Henry Wellcome Fellowship [201374/Z/16/Z] and a UKRI FLF [MR/T018119/1]; a National Institute for Health and Care Research (NIHR) Advanced Fellowship [NIHR3016640] to L.S., and by core funding from the Wellcome/EPSRC Centre for Medical Engineering [WT203148/Z/16/Z]; and by the NIHR Biomedical Research Centre based at Guy's and St Thomas' National Health Service (NHS) Foundation Trust and King's College London and/or the NIHR Clinical Research Facility.

## Supporting information


**Figure S1:** Cohort characteristics for the PPROM subjects (blue) and the control subjects (red) depicting the distribution of birth weight centile, gestational ag @ delivery, time between rupture of the membranes and scan and time between scan and delivery.
**Table S1:** Patient cohort characteristics for the control and the PPROM cohort. Significant results (*P* < 0.005) are colored in blue.

## Data Availability

Access to the data is available to all academic parties by email to the corresponding author in line with the ethical approval of this study. The code used for the analysis is available under https://github.com/Jamahu/pypla.git.
