# Supplementary material for: Multi‐modal MRI reveals changes in placental function following preterm premature rupture of membranes
Source: Magn Reson Med. 2022 Oct 18;89(3):1151–9. doi: 10.1002/mrm.29483 (PMC10091779; doi:10.1002/mrm.29483)
Supplement: Supplementary file 1 — Figure S1: Cohort characteristics for the PPROM subjects (blue) and the control subjects (red) depicting the distribution of birth weight centile, gestational ag @ delivery, time between rupture of the membranes and scan and time between scan and delivery. Table S1: Patient cohort characteristics for the control and the PPROM cohort. Significant results (P < 0.005) are colored in blue. [file MRM-89-1151-s001.docx]

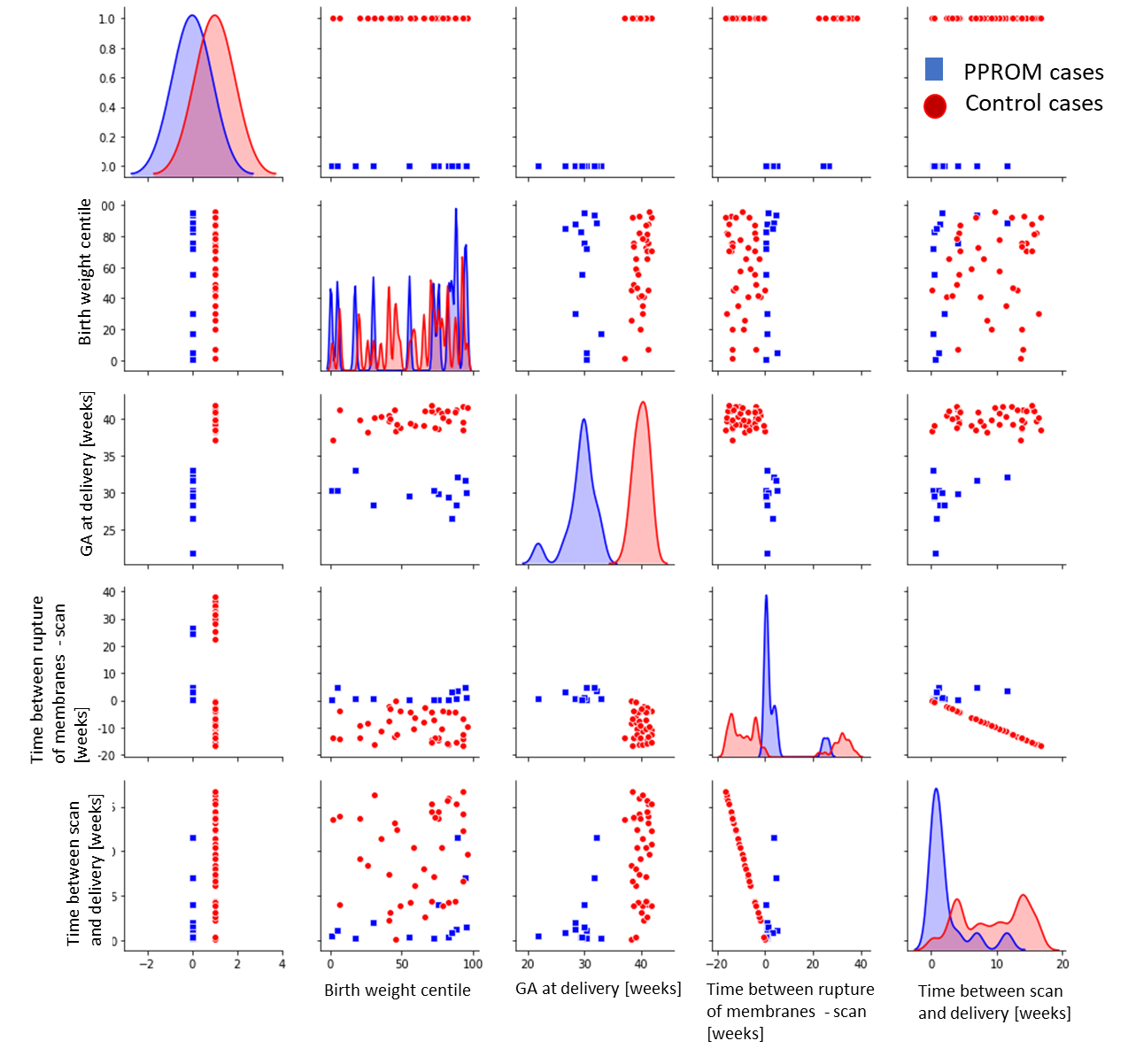


***Supporting Information Figure S1:*** *Cohort characteristics for the PPROM subjects (blue) and the control subjects (red) depicting the distribution of birth weight centile, GA @ delivery, time between rupture of the membranes and scan and time between scan and delivery*

*.*

| **N=76** | **Control (N=60)** | **PPROM (N=16)** |  |
| --- | --- | --- | --- |
| **Gestational age @ scan [weeks]** | 30.71 +- 4.63 | 26.77 +- 3.17 | p>0.5 |
| **Body mass index [kg/m2]** | 21.54 +- 2.55 | 24.13 +- 2.68 | p>0.5 |
| **Age [years]** | 35.38 +- 3.49 | 35.56 +- 4.32 | p>0.5 |
| **Gestational age @ delivery [weeks]** | 40.03 +- 1.16 | 29.39 +- 2.61 | **p<0.05*** |
| **Birth weight centile** | 59.38 +- 26.39 | 60.83 +- 33.77 | p>0.5 |
| **Time rupture of the membranes-scan [weeks]** | NA | 4.52 +- 8.05 | NA |
| **Time scan-delivery [weeks]** | 9.34 +- 4.91 | 2.28 +- 3.12 | **p<0.05*** |

***Supporting Information Table S1:*** *Patient cohort characteristics for the control and the PPROM cohort.*
